# Weighted Color Image Encryption Algorithm Based on RNA Extended Dynamic Coding and Quantum Chaotic System

**DOI:** 10.3390/e27080852

**Published:** 2025-08-11

**Authors:** Xiangyu Zhang, Heping Wen, Wei Feng, Shenghao Kang, Zhiyu Xie, Xuexi Zhang, Yiting Lin

**Affiliations:** 1 School of Automation, Guangdong University of Technology, Guangzhou 510006, China; 2112304329@mail2.gdut.edu.cn (X.Z.); zhangxuexi928@gmail.com (X.Z.); 2 School of Electronic Information, Zhongshan Institute, University of Electronic Science and Technology of China, Zhongshan 528402, China; 13929699132@163.com (S.K.); dr.zhiyuxie@gmail.com (Z.X.); yitinglin@zsc.edu.cn (Y.L.); 3 School of Mathematics and Computer Science, Panzhihua University, Panzhihua 617000, China; fengwei@pzhu.edu.cn

**Keywords:** RNA extended, color image encryption, dynamic coding, chaotic system

## Abstract

The rapid development of Internet technology, while providing convenient services for users, has also aroused deep concern among the public about the issue of privacy leakage during image data transmission. To address this situation, this article proposes a color image encryption algorithm based on RNA extended dynamic coding and quantum chaos (CIEA-RQ). This algorithm significantly improves the ability of the system to withstand cryptographic attacks by introducing RNA extended dynamic encoding with 384 encoding rules. The employed quantum chaotic map improves the randomness of chaotic sequences and increases the key space. First, the algorithm decomposes the plaintext image into bit planes and obtains two parts, high 4-bit and low 4-bit planes, based on different weights of information. Then, the high 4-bit planes are partitioned into blocks and scrambled, and the scrambled planes are confused using RNA extended coding rules. Meanwhile, the low 4-bit planes employ a lightweight XOR operation to improve encryption efficiency. Finally, the algorithm performs cross-iterative diffusion on the processed high 4-bit and low 4-bit planes and then synthesizes a color ciphertext image. Experimental simulations and security assessments demonstrate the superior numerical statistical outcomes of the CIEA-RQ. According to the criteria of cryptanalysis, it can effectively resist known-plaintext attacks and chosen-plaintext attacks. Therefore, the CIEA-RQ presented in this article serves as an efficient digital image privacy safeguard technique, promising extensive applications in image secure transmission for the upcoming generation of networks.

## 1. Introduction

As the Internet becomes increasingly popular, the transmission frequency of information on public network media has gradually become higher [1,2,3]. Digital images are widely used as simple and intuitive carriers that carry a large amount of visual information [4,5,6]. However, images are easily damaged and leaked when transmitted over the Internet, causing an incalculable loss and damage to users [7,8,9,10]. Therefore, the safeguarding of images has become the focus of attention, especially in areas such as personal privacy, business, and the military that require high confidentiality [11,12,13,14]. Images possess characteristics of high information content, substantial redundancy, and strong correlation [15,16,17,18]; this makes traditional text encryption methods like IDEA, DES, and AES less suitable for encrypting digital images [19,20,21,22]. In this context, chaos has garnered significant attention due to its characteristics of exhibiting behavior akin to randomness [23,24,25,26]. Chaotic systems exhibit remarkable sensitivity to starting conditions, leading to distinct trajectories for different initial states, ultimately generating unpredictable chaotic signals [27,28,29,30]. This property gives chaos a natural advantage in the field of cryptography; compared with the traditional text encryption method, it has a stronger scrambling effect on image encryption and can obtain a more excellent encryption effect. Therefore, it is considered to be a promising new method in digital image encryption.

In recent years, the international academic community has conducted in-depth research on the impact of chaotic systems and algorithms on the security of encryption systems. In 2022, Wang et al. [31] suggested a bit-level and pixel-level image encryption scheme combining two chaotic systems. This scheme proves to have better security. In 2023, Wang et al. [32] devised an innovative approach for encrypting images based on a two-dimensional sine-embedded coupling map that consist of bit-level cross-transformation and efficient diffusion processing. The designed algorithm is able to withstand common types of attacks. Besides the above-mentioned studies, the academic community has also explored the possibility of combining image encryption with biological coding techniques. Wu et al. [33] proposed a DNA extension code with 3-bit binary streams for downlink data encryption in an orthogonal frequency division multiplexing passive optical network. The scheme increases security while having the ability to resist fiber nonlinearity and optical channel response. Jasra et al. [34] suggested a new algorithm for encrypting images adopting elliptic curve cryptography, hyperchaotic systems, and dynamic DNA encoding. Results and analysis reveal that this method is computationally efficient and has strong robustness. Singh et al. [35] presented a secure model encryption algorithm on images for Industrial Internet of Things incorporating DNA cryptography and chaos, demonstrating resilience against various attacks. In 2024, Wang et al. [36] designed a novel chaotic two-dimensional hyperchaotic exponential adjusted Logistic and sine map, which combines a dynamic diffusion strategy and RNA operation to achieve high security of ciphertext images. The experimental results prove the feasibility of the proposed algorithm. In 2025, a novel Hénon nonlinear coupled mapping lattice for spatiotemporal chaos was proposed by Yang et al. [37]. Image encryption is achieved by internal diffusion and RNA encoding operations. The experimental results show that the algorithm has high security.

Although existing chaotic encryption has made great progress, some algorithms cannot resist the chosen-plaintext attack, and the scrambling mechanism is not complex enough. Hence, we carried out this work from the perspective of security enhancement. In this article, the CIEA-RQ is proposed. Firstly, the color plaintext image is segmented into bit planes according to the information weights to obtain the high 4-bit and low 4-bit planes. Secondly, the hash function is employed to produce the key associated with the plaintext image as an input to the chaotic system to obtain chaotic sequences for block scrambling and RNA extended dynamic coding of the high 4-bit planes. Next, a lightweight XOR operation is performed on the low 4-bit planes by the chaotic sequence. Finally, the high 4-bit and low 4-bit planes are cross-iteratively diffused and synthesized to obtain a color ciphertext image. In block scrambling, the image is divided into multiple three-dimensional (3-D) submatrices of the same size and then encrypted. Compared with the two-dimensional (2-D) matrix, the dimension of image encryption is improved, the encryption correlation between layers is enhanced, separate encryption between bit planes is avoided, and the security is improved. Secondly, considering the information weight difference represented by each bit in the pixel value, the image is divided into high 4-bit planes and low 4-bit planes, and differential encryption strategies are adopted to achieve a better balance between security and efficiency. Compared with the common RNA coding rules in the existing literature, this paper significantly improves the diversity and randomness of pixel diffusion by extending the RNA bases and effectively improves the anti-attack ability of the system. The hierarchical encryption strategy, XOR operation, and coding operation proposed in this paper are easy to implement and are suitable for image protection in the Internet of Things. In addition, the algorithm has good modular characteristics, which can cut and optimize the encryption process according to the application requirements, so it has strong feasibility and applicability in actual deployment.

This paper’s primary contributions and innovations can be outlined as follows:

1. The algorithm introduces a new RNA extended coding mechanism with 384 coding rules, far exceeding the 8 of the traditional method, which greatly enhances the randomness and dynamic characteristics of the encryption and effectively enhances the algorithm’s resistance against various types of attacks.
2. The algorithm employs bit plane processing based on information weights to differentially encrypt the high 4-bit and low 4-bit planes, effectively balancing the security and efficiency performance of CIEA-RQ.
3. In this article, the hash function, RNA extended dynamic coding, high- and low-bit hierarchical encryption, and the cross-iterative diffusion mechanism are organically combined, and multi-level diffusion is used to enhance the resistance of the algorithm to various attacks.
4. The quantum Logistic map used in this paper not only has improved nonperiodicity and randomness but also has multiple initial values and control parameters, leading to an enlarged key space for the system.

The rest of this article is structured as follows. Section 2 introduces the quantum Logistic map, RNA extended dynamic coding rules, and operation methods. Section 3 presents the CIEA-RQ devised in this work. Section 4 covers the simulation test as well as the analysis. The last section provides the summation of this article.

## 2. Related Theory

### 2.1. Quantum Logistic Map

Chaotic systems are frequently employed in image processing owing to their randomness and remarkable sensitivity. One-dimensional chaotic systems have a simple structure and the generated orbits are easily predictable. The traditional Logistic chaotic map equation is articulated as follows:

(1) $$x_{n+1}=\mu x_{n}\left(1-x_{n}\right),$$

where the control parameter μ∈(3.5699,4], $x_{n}\in \left(0,1\right)$.

Goggin et al. [38] used the recoil rotor model to quantize the classical Logistic chaotic system and produce the corresponding quantum Logistic map. This chaotic map adds control parameters and includes a non-vanishing disturbance quantity at the end, which enhances the nonperiodicity and randomness of the system. It can be expressed as follows:

(2) $$\left\{\begin{array}{c} x_{n+1}=r\left(x_{n}-{\left|x_{n}\right|}^{2}\right)-ry_{n},\\ y_{n+1}=-y_{n}e^{-2\beta }+e^{-\beta }r\left[\left(2-x_{n}-x_{n}^{*}\right)y_{n}-x_{n}z_{n}^{*}-x_{n}^{*}z_{n}\right],\\ z_{n+1}=-z_{n}e^{-2\beta }+e^{-\beta }r\left[2\left(1-x_{n}^{*}\right)z_{n}-2x_{n}y_{n}-x_{n}\right], \end{array}\right.$$

where $x_{n}$, $y_{n}$, and $z_{n}$ are the state values of the system, $x_{n}^{*}$ and $z_{n}^{*}$ are the complex conjugates of $x_{n}$ and $z_{n}$, respectively,  β is a dissipative parameter, and *r* is an adjustable parameter. When the system parameters r∈[0,4], β≥6, and the state values $x_{n}\in \left[0,1\right]$, $y_{n}\in \left[0,0.1\right]$, and $z_{n}\in \left[0,0.2\right]$, the system is in a chaotic state.

### 2.2. RNA Extended Dynamic Coding

RNA is a chain-like molecule that grows from ribonucleotides condensed by a phosphodiester bond. A ribonucleotide molecule consists of phosphate, ribose, and bases. In RNA, there are four types of bases, A, U, G, and C, with A-U and C-G as complementary base pairs. To enhance encryption randomness, four new bases, M, N, S, and W, are extended from the original bases, and these new bases follow specific pairing rules, i.e., M is paired with W and S is paired with N. Also, in binary, 0 and 1 are complementary and so are 000-111, 001-110, 010-101, and 011-100. Using base pairs to encode numbers, there are a total of 384 coding rules, as presented in Table 1, thus greatly improving the randomness of the matching. The operation between bases is realized by addition table, subtraction table or XOR table, as listed in Table 2, Table 3 and Table 4, respectively.

## 3. Proposed CIEA-RQ

**Figure 1:**
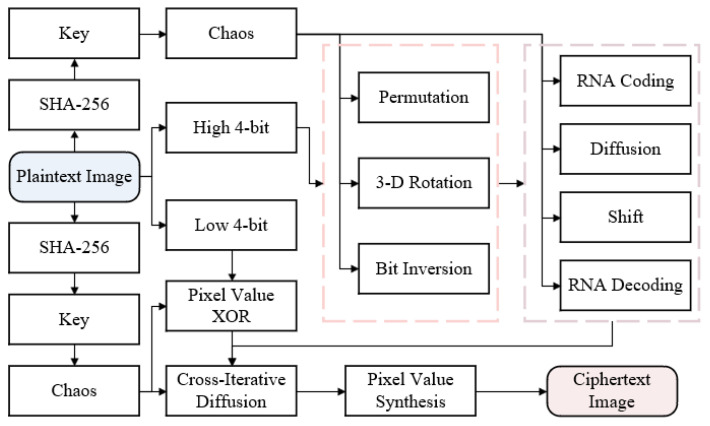
Flow diagram of the CIEA-RQ.

The three parts included in the CIEA-RQ are described in detail as follows. The first part is key generation. The key associated with the plaintext image is produced using SHA-256. The second part is the image encryption process. The precise encryption procedure consists of two main modules. The first module is the high 4-bit plane and low 4-bit plane encryption after pixel value segmentation, and the second module is the cross-iterative diffusion of high 4-bit and low 4-bit planes. The third part is the image decryption process, where the decryption order is the reverse of the encryption order, and each step in the decryption process is an encryption inverse operation. Figure 1 depicts a flow diagram of the CIEA-RQ.

### 3.1. Key Generating

The pixel values of the plaintext image are applied as input to SHA-256, which outputs fixed 256 bits. The 256 bits are split into thirty-two 8-bit groups, each converted into a decimal number, resulting in 32 decimal numbers, each of which can be expressed as $h_{i}\in \left\{0,1,\ldots ,255\right\}$, where i=[1,2,…,32]. The specific procedure is defined as follows:

(3) $$\left\{\begin{array}{c} x_{0}\left(i\right)=x^{\prime }+\left(h_{i}⊕h_{i+1}⊕h_{i+2}⊕h_{i+3}⊕h_{i+4}\right)/10,000,\\ y_{0}\left(i\right)=y^{\prime }+\left(h_{i+8}⊕h_{i+9}⊕h_{i+10}⊕h_{i+11}⊕h_{i+12}\right)/10,000,\\ z_{0}\left(i\right)=z^{\prime }+\left(h_{i+16}⊕h_{i+17}⊕h_{i+18}⊕h_{i+19}⊕h_{i+20}\right)/10,000,\\ r\left(i\right)=r^{\prime }+\left(h_{i+4}⊕h_{i+9}⊕h_{i+14}⊕h_{i+19}⊕h_{i+24}\right)/100,000,\\ \beta \left(i\right)=\beta^{\prime }+\left(h_{i}⊕h_{i+6}⊕h_{i+12}⊕h_{i+18}⊕h_{i+24}\right)/100,000, \end{array}\right.$$

where i=[1,2,…,8], $x^{\prime }$, $y^{\prime }$, $z^{\prime }$, $r^{\prime }$, $\beta^{\prime }$ are given initial values, $x_{0}\left(i\right)$, $y_{0}\left(i\right)$, $z_{0}\left(i\right)$, r(i), and β(i) are the keys of the quantum Logistic map, and ⊕ is the XOR operation.

### 3.2. Encryption Process

In the realm of image manipulation, given that each pixel point is meticulously divided into a depth of 8 bits, the high 4 bits tend to contribute more to the visual information, and therefore implementing more sophisticated encryption measures for these bits can effectively resist potential attacks. In comparison, the low 4 bits have a much smaller impact on the overall visual effect of the image than the high 4 bits. For the low 4 bits, the choice of lightweight encryption means a certain level of security can be maintained and avoids unnecessary overhead, thus realizing a clever balance between encryption efficiency and strength. Using a 3-D matrix *P* with a size of M×N×3 as an illustration, the specific procedure is outlined below.

#### 3.2.1. Sequence Generation and Preprocessing

Using the keys $x_{0}\left(i\right)$, $y_{0}\left(i\right)$, $z_{0}\left(i\right)$, r(i), and β(i) as input parameters to the chaos equation, the pseudo-random sequences $S_{1}$, $S_{2}$, $S_{3}$, $S_{4}$, $S_{5}$, $S_{6}$, $S_{7}$, and $S_{8}$ are generated, and then the pseudo-random sequences are transformed to obtain the final desired sequences $S_{1}^{\prime }$, $S_{2}^{\prime }$, $S_{3}^{\prime }$, $S_{4}^{\prime }$, $S_{5}^{\prime }$, $S_{6}^{\prime }$, $S_{7}^{\prime }$, and $S_{8}^{\prime }$, with the lengths of 3MN/16, 9MN/16, 12MN, 4MN, 8MN, MN, 3MN, and 6MN, respectively. The operation is shown as follows:

(4) $$\left\{\begin{array}{c} S_{1}^{\prime }=floor\left(\mathrm{mod}\left(S_{1}\times 10^{10},3MN/16\right)\right)+1,\\ S_{2}^{\prime }=floor\left(\mathrm{mod}\left(S_{2}\times 10^{10},4\right)\right),\\ S_{3}^{\prime }=floor\left(\mathrm{mod}\left(S_{3}\times 10^{10},2\right)\right),\\ S_{4}^{\prime }=floor\left(\mathrm{mod}\left(S_{4}\times 10^{10},3\right)\right),\\ S_{5}^{\prime }=floor\left(\mathrm{mod}\left(S_{5}\times 10^{10},384\right)\right)+1,\\ S_{6}^{\prime }=floor\left(\mathrm{mod}\left(S_{6}\times 10^{10},8\right)\right),\\ S_{7}^{\prime }=floor\left(\mathrm{mod}\left(S_{7}\times 10^{10},16\right)\right),\\ S_{8}^{\prime }=floor\left(\mathrm{mod}\left(S_{8}\times 10^{10},16\right)\right), \end{array}\right.$$

where mod(·) denotes the modulo operation function and floor(*x*) returns the greatest integer less than or equal to x.

#### 3.2.2. High 4-Bit Plane and Low 4-Bit Plane Encryption

Step1: Bit decomposition

After extracting the high 4 bits of matrix *P*, decomposition is performed to form a 3-D matrix $C_{A1}$ of M×N×12.

Step2: High 4-bit plane block scrambling

**Figure 2:**
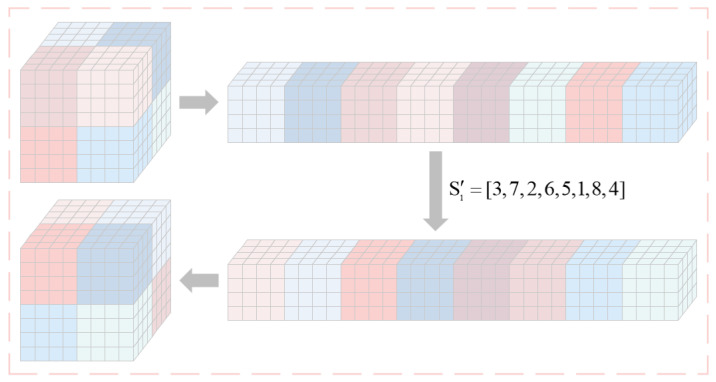
Flow diagram of the proposed block permutation.

The matrix $C_{A1}$ is divided into blocks, with each submatrix having a size of 4×4×4. Then these submatrices are recombined sequentially into a row to obtain the submatrix sequence $I_{1}\left(i\right)$, i=[1,2,…,3MN/16]. $S_{1}^{\prime }$ is used to permute $I_{1}\left(i\right)$; the operation is detailed as follows:

(5) $$\left\{\begin{array}{c} I_{2}=I_{1}\left(i\right),\\ I_{1}\left(i\right)=I_{1}\left(S_{1}^{\prime }\left(i\right)\right),\\ I_{1}\left(S_{1}^{\prime }\left(i\right)\right)=I_{2}, \end{array}\right.$$

where $I_{2}$ is the intermediate variable. Then the sequence of submatrices is sequentially recombine into the matrix $C_{A1}$. Using a matrix of 8×8×8 as an example, Figure 2 illustrates the flow diagram for the specific block permutation.

**Figure 3:**
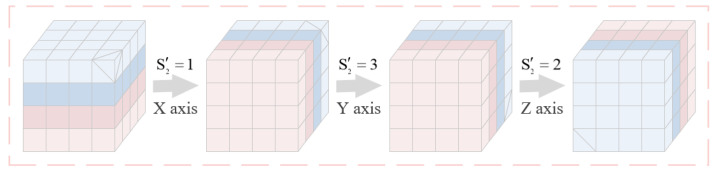
Flow diagram of the proposed block rotation.

The submatrices after the block permutation are rotated, and the number of times each submatrix is rotated $90^{\circ }$ counterclockwise around three axes is determined by sequence $S_{2}^{\prime }$ to obtain the rotated matrix $C_{A2}$. Using the rotation of a 4×4×4 submatrix as an example, the flow diagram of the block rotation is represented in Figure 3. Finally the matrix $C_{A2}$ is inverted by the sequence $S_{3}^{\prime }$ to obtain an inverted matrix $C_{A3}$. The detailed inverse operation is shown below:

(6) $$\left\{\begin{array}{cc} C_{A3}\left(i\right)=1-C_{A2}\left(i\right), & if\;S_{3}^{\prime }=0,\\ C_{A3}\left(i\right)=C_{A2}\left(i\right), & if\;S_{3}^{\prime }=1, \end{array}\right.$$

where i=[1,2,…,12MN].

Step3: High 4-bit plane RNA extended dynamic coding diffusion

All the pixel values of the matrix $C_{A3}$ from bottom to top are combined into one octal number for every three binary numbers. The coding rules are chosen based on the first half of the sequence $S_{5}^{\prime }$, and then each octal number is encoded to obtain a matrix $C_{A4}$ with dimension M×N×4. The sequence $S_{6}^{\prime }$ is recombined into a 2-D matrix of dimension M×N, which is encoded in the same way as the corresponding position of the first layer of the matrix $C_{A4}$, and after encoding, the matrix $I_{3}$ is obtained. Using the sequence $S_{4}^{\prime }$ to select one of addition, subtraction, or XOR to perform the operation, the matrix $C_{A4}$ is iteratively diffused to obtain matrix $C_{A5}$. The specific operation is shown as follows:

(7) $$\left\{\begin{array}{cc} C_{A5}\left(i,j,k\right)=C_{A4}\left(i,j,k\right)\Theta I_{3}\left(i,j\right), & if\;k=1,\\ C_{A5}\left(i,j,k\right)=C_{A4}\left(i,j,k\right)\Theta C_{A5}\left(i,j,k-1\right), & if\;k=2∼4, \end{array}\right.$$

where *k* denotes the number of layers, i=[1,2,…,M], j=[1,2,…,N], and Θ indicates addition, subtraction, or XOR operation, with the specific choices being controlled by sequence $S_{4}^{\prime }$.

Step4: High 4-bit plane RNA extended dynamic coding shift

**Figure 4:**
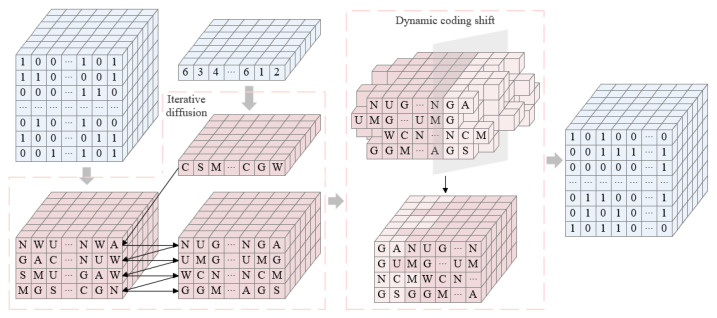
Flow diagram of the RNA extended dynamic coding encryption.

For the matrix $C_{A5}$, according to the coding rules corresponding to the sequence $S_{5}^{\prime }$, the next row is shifted to the right according to the last pixel value of the previous row, and the matrix $C_{A6}$ after the shift is obtained. The second half of the sequence $S_{5}^{\prime }$ is selected for decoding. The decoded matrix is synthesized into a pixel value every 4 bits from bottom to top, and a 3-D matrix $A_{1}$ of dimension M×N×3 is obtained. The flow diagram of the RNA extended dynamic coding encryption is shown in Figure 4. The specific shift operation is shown as follows:

(8) $$\left\{\begin{array}{cc} I_{4}\left(1:num_{i-1}\right)=C_{A5}\left(i,N-\mathrm{nu}m_{i-1}+1:N,k\right),\\ I_{4}\left(num_{i-1}+1:N\right)=C_{A5}\left(i,1:N-num_{i-1},k\right),\; & if\;i=2∼M,\\ C_{A6}\left(i,:,k\right)=I_{4},\\ I_{4}\left(1:num_{M}\right)=C_{A5}\left(i,N-num_{M}+1:N,k\right),\\ I_{4}\left(num_{M}+1:N\right)=C_{A5}\left(i,1:N-num_{M},k\right),\; & if\;i=1,\\ C_{A6}\left(i,:,k\right)=I_{4}, \end{array}\right.$$

where *k* denotes the number of layers, k=[1,2,3,4], $num_{i-1}$ signifies the last pixel value of the row i−1, $num_{M}$ denotes the last pixel value of the row *M*, and $I_{4}$ is the intermediate variable.

Step5: Low 4-bit plane XOR operation

The low 4 bits of matrix *P* are extracted but not decomposed to form a 3-D matrix $B_{1}$ of dimension M×N×3. The sequence $S_{7}^{\prime }$ is converted into a matrix of size M×N×3, and then it performs the XOR operation with matrix $B_{1}$ to obtain matrix $B_{2}$.

#### 3.2.3. Pixel Value Cross-Iteration Diffusion

Matrices $A_{1}$ and $B_{2}$ are recombined into vectors. The sequence $S_{8}^{\prime }$ is used to perform the add and modulo operation on vectors $A_{1}$ and $B_{2}$, and finally the pixel values are synthesized to acquire the ciphertext image *C*. Algorithm 1 gives the pseudo-code of pixel value cross-iterative diffusion.
**Algorithm 1:** Pixel value cross-iterative diffusion.**Input:** Vectors $A_{1}$ and $B_{2}$ of length 3MN, sequence $S_{8}^{\prime }$ with length 6MN**Output:** Ciphertext image C 1:**if** i=1 **then**  2:      $A_{2}\left(i\right)\leftarrow \left(A_{1}\left(3MN\right)+B_{2}\left(i\right)+S_{8}^{\prime }\left(i\right)\right)\;\mathrm{mod}\;16$ 3:**else if** *i* from 2 to 3MN **then** 4:      $A_{2}\left(i\right)\leftarrow \left(A_{1}\left(i-1\right)+B_{2}\left(i\right)+S_{8}^{\prime }\left(i\right)\right)\;\mathrm{mod}\;16$ 5:**end if** 6:**if** i=1 **then** 7:      $B_{3}\left(i\right)\leftarrow \left(B_{2}\left(3MN\right)+A_{2}\left(i\right)+S_{8}^{\prime }\left(3MN+i\right)\right)\;\mathrm{mod}\;16$ 8:**else if** *i* from 2 to 3MN **then** 9:      $B_{3}\left(i\right)\leftarrow \left(B_{2}\left(i-1\right)+A_{2}\left(i\right)+S_{8}^{\prime }\left(3MN+i\right)\right)\;\mathrm{mod}\;16$10:**end if**11:convert the vectors $A_{2}$ and $B_{3}$ into M×N×3 3-D matrices $A_{3}$ and $B_{4}$12:$C\leftarrow A_{3}\times 2^{4}+B_{4}$

### 3.3. Decryption Process

Performing the opposite operation on the encryption procedure yields the decrypted image. To decrypt the image, it is essential to securely transmit the key to the decryption end via a secure channel. In the image encryption stage, we first perform pixel value segmentation on the image, then encrypt the high 4-bit and the low 4-bit planes, respectively, and finally perform pixel values synthesis after cross-iterative diffusion. Therefore, in the decryption stage, we first perform pixel value segmentation. Secondly, the two segmented matrices are reversely iteratively diffused and then decrypted separately. Finally, the decrypted image is synthesized.

## 4. Experimental Results and Analysis Discussion

We conducted experiments on a PC furnished with MATLAB R2022b software. The PC is powered by a 13th Gen Intel(R) Core(TM) i7-13650HX 2.60 GHz processor, with 16 GB of RAM and a 1TB hard disk. The operating system used is Windows 11. The experiment employed images from the USC-SIPI dataset (http://sipi.usc.edu/database/ (accessed on 15 June 2025)).

### 4.1. Encryption and Decryption Experiments

**Figure 5:**
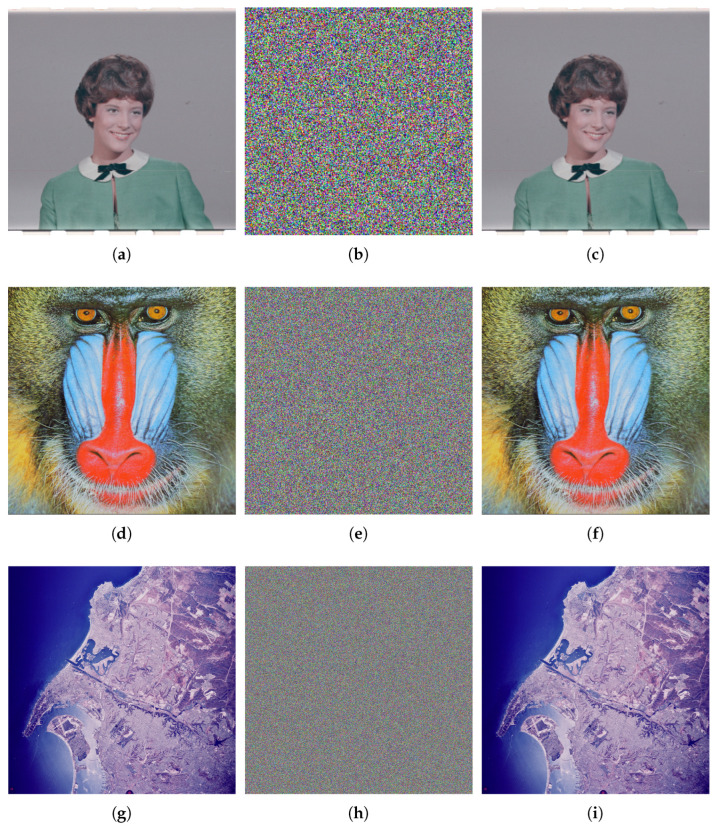
Encryption and decryption results. (**a**) Plaintext image 4.1.03. (**b**) Ciphertext image of (**a**). (**c**) Decrypted image of (**b**). (**d**) Plaintext image 4.2.03. (**e**) Ciphertext image of (**d**). (**f**) Decrypted image of (**e**). (**g**) Plaintext image 2.2.01. (**h**) Ciphertext image of (**g**). (**i**) Decrypted image of (**h**).

Figure 5 reveals the encryption and decryption results of the CIEA-RQ. It can be observed that the ciphertext image presents noise-like characteristics, which makes it difficult to identify any original content and effectively prevents attackers from obtaining useful information. After decryption with the correct key, the image can be completely restored without visual information loss. The experimental results verify the effectiveness of CIEA-RQ in image confidentiality and reversibility.

### 4.2. Histogram Analysis

**Figure 6:**
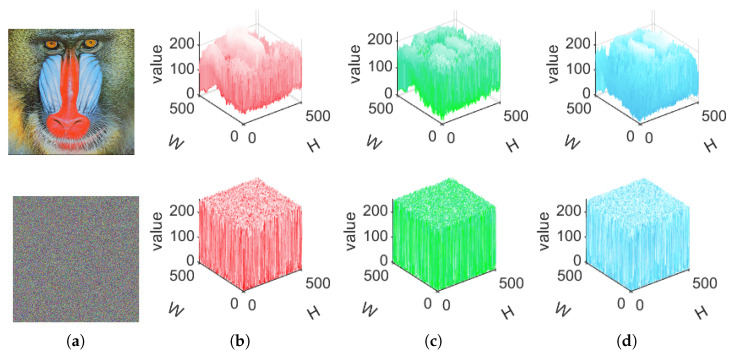
Three-dimensional visualization of the image before and after encryption. (**a**) Plaintext image and ciphertext image. (**b**) Red channel. (**c**) Green channel. (**d**) Blue channel.

The histogram portrays the occurrence frequency of individual pixel values within an image. A robust security scheme yields a ciphertext image histogram that is entirely different from the plaintext image histogram, and the ciphertext image should exhibit an near-uniform distribution of pixel values. In this experiment, we encrypt a color image of size 512×512×3 and perform histogram experimental analysis. Figure 6 illustrates the simulation result. Notably, the ciphertext image’s histogram diverges from that of the plaintext image, displaying an almost uniform distribution of pixel values.

### 4.3. Adjacent Pixel Correlation Analysis

**Figure 7:**
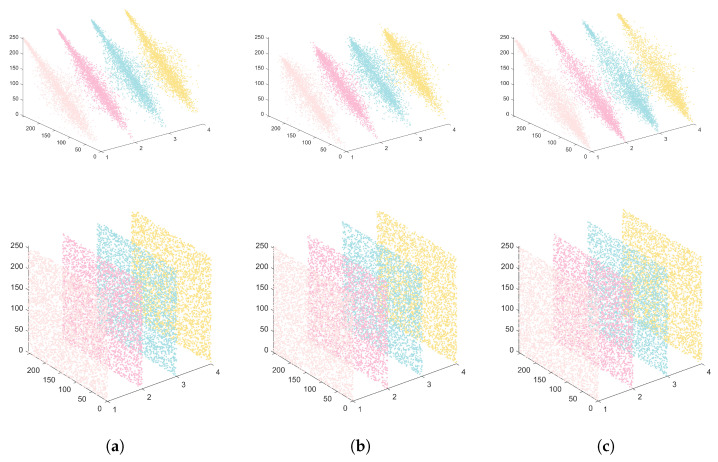
Simulation results of adjacent pixels before and after image encryption. (**a**) Horizontal/vertical/diagonal/anti-diagonal for red channel of 4.2.03. (**b**) Horizontal/vertical/diagonal/anti-diagonal for green channel of 4.2.03. (**c**) Horizontal/vertical/diagonal/anti-diagonal for blue channel of 4.2.03.

To assess the degree of correlation, we randomly chose 3000 pairs of adjacent pixels from both the plaintext and ciphertext images for calculation. In the plaintext image, the differences between them are small and have a high correlation in horizontal, vertical, diagonal, and anti-diagonal directions. Nonetheless, superior algorithms can effectively disrupt this correlation, resulting in an insignificant correlation between adjacent pixels in the ciphertext image. The correlation coefficient between two adjacent pixel sequences *X* and *Y* can be formulated as follows:

(9) $$C\left(x,y\right)=\frac{\frac{1}{N}\sum_{i=1}^{N}\left(x_{i}-E\left(x\right)\right)\left(y_{i}-E\left(y\right)\right)}{\sqrt{\frac{1}{N}\sum_{i=1}^{N}{\left(x_{i}-E\left(x\right)\right)}^{2}}\sqrt{\frac{1}{N}\sum_{i=1}^{N}{\left(y_{i}-E\left(y\right)\right)}^{2}}},$$

where E(x) and E(y) denote the mathematical expectations of *X* and *Y*. In order to more clearly and intuitively display the extent of change in image correlation before and after encryption, we chose two forms to present the experimental results. Figure 7 displays the analysis of the correlation in different directions. Table 5 displays the correlation coefficients’ outcomes pertaining to the images before and after encryption, and Table 6 shows the results of CIEA-QR compared with other algorithms.

### 4.4. Differential Attack Analysis

As a widely used cryptanalysis method, the effectiveness of differential attacks relies on the sensitivity of the algorithm to small input differences. To accurately evaluate the robustness of an image encryption scheme against differential attacks, the number of pixels change rate (NPCR) and the unified average changing intensity (UACI) are commonly applied as test metrics. Assuming that two ciphertext images, $C_{1}$ and $C_{2}$, are the results of encrypting two plaintext images by just a one-bit difference, the NPCR and UACI between $C_{1}$ and $C_{2}$ are calculated as follows:

(10) $$\left\{\begin{array}{c} NPCR\left(C_{1},C_{2}\right)=\sum_{i=1}^{M}\sum_{j=1}^{N}\frac{W(i,j)}{H}\times 100\%,\\ UACI\left(C_{1},C_{2}\right)=\sum_{i=1}^{M}\sum_{j=1}^{N}\frac{|C_{1}\left(i,j\right)-C_{2}\left(i,j\right)|}{H\times Q}\times 100\%, \end{array}\right.$$

(11) $$W\left(i,j\right)=\left\{\begin{array}{cc} 1, & C_{1}\left(i,j\right)\neq C_{2}\left(i,j\right),\\ 0, & C_{1}\left(i,j\right)=C_{2}\left(i,j\right), \end{array}\right.$$

where *Q* represents the maximum value that a pixel in the image can reach, the total count of pixels is represented by *H*, and [M,N] indicates the image size.

Wu et al. [42] have established a rigorous set of NPCR and UACI testing standards to comprehensively assess the performance of encryption. For an ideal encryption algorithm, NPCR should exceed a predetermined threshold, while the UACI needs to fall within a specific range. The calculation of the threshold $N_{\alpha }^{*}$ for the NPCR test and the range $\left(U_{\alpha }^{*-},U_{\alpha }^{*+}\right)$ for the UACI test can be summarized as follows:

(12) $$N_{\alpha }^{*}=\frac{Q-\Phi^{-1}\left(\alpha \right)\sqrt{Q/H}}{Q+1},$$

(13) $$\left\{\begin{array}{c} U_{\alpha }^{*-}=\mu_{u}-\Phi^{-1}\left(\alpha /2\right)\sigma_{u},\\ U_{\alpha }^{*+}=\mu_{u}+\Phi^{-1}\left(\alpha /2\right)\sigma_{u}, \end{array}\right.$$

(14) $$\mu_{u}=\frac{Q+2}{3Q+3},$$

(15) $$\sigma_{u}^{2}=\frac{\left(Q+2\right)\left(Q^{2}+2Q+3\right)}{18{\left(Q+1\right)}^{2}QH},$$

where $\Phi^{-1}\left(•\right)$ is the inverse cumulative density function of the standard normal distribution. With the significance level α set at 0.05 in this article, the testing criteria for different image sizes are as follows: for 256×256 images, $N_{\alpha }^{*}$ is 99.5693% and the $\left(U_{\alpha }^{*-},U_{\alpha }^{*+}\right)$ range is (33.2824%, 33.6447%); for 512×512 images, $N_{\alpha }^{*}$ is 99.5893% and the $\left(U_{\alpha }^{*-},U_{\alpha }^{*+}\right)$ range is (33.3730%, 33.5541%); and for 1024×1024 images, $N_{\alpha }^{*}$ is 99.5994% and the $\left(U_{\alpha }^{*-},U_{\alpha }^{*+}\right)$ range is (33.4183%, 33.5088%). The encryption results for images of different sizes are displayed in Table 7. The empirical outcomes suggest that the CIEA-RQ is resistant to differential attacks.

### 4.5. Information Entropy Analysis

Information entropy is an important metric for evaluating the distribution of gray values in an image and gauging the randomness of image information. It is specifically described as follows:

(16) $$H\left(x\right)=-\sum_{i=0}^{2^{F}-1}p(x_{i})\log_{2}p\left(x_{i}\right),$$

where $p(x_{i})$ represents the frequency of information occurrence, and for an 8-bit grayscale image, F equals 8. The information entropy’s theoretical value stands at 8. When the outcome approaches 8, it signifies a great level of randomness in the image’s grayscale value distribution and reduces the likelihood of information leakage. In accordance with the experimental test outcomes as presented in Table 8, the algorithm shows higher randomness and lower information leakage probability, thus effectively resisting statistical analysis attacks.

### 4.6. Robustness Analysis

During the decryption process, some noise interference and data loss can affect the decryption effect. This section will introduce salt-and-pepper noise and cropping attacks to the ciphertext image, testing the scheme’s resistance to noise pollution and data loss during decryption and analyzing its overall robustness.

#### 4.6.1. Salt-and-Pepper Noise Analysis

**Figure 8:**
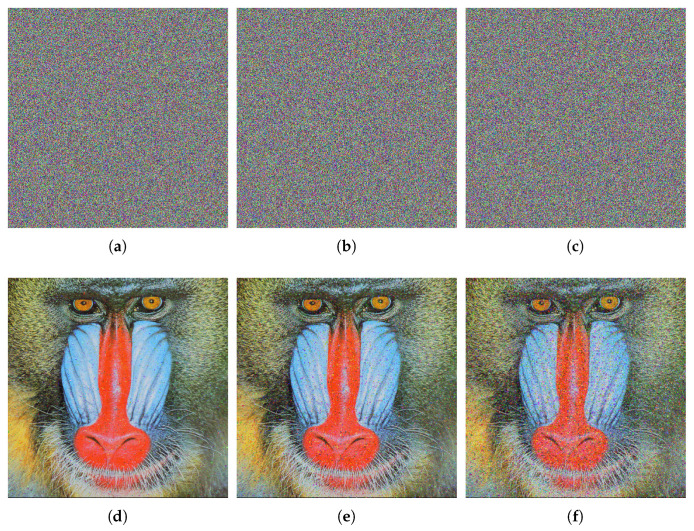
Salt-and-pepper noise attack detection for ciphertext image of 4.2.03. (**a**) Salt-and-pepper noise (0.005). (**b**) Salt-and-pepper noise (0.01). (**c**) Salt and-pepper noise (0.03). (**d**) Decrypted image of (**a**). (**e**) Decrypted image of (**b**). (**f**) Decrypted image of (**c**).

In the decryption process, salt-and-pepper noise with intensities of 0.005, 0.01, and 0.03 is introduced to the ciphertext image, followed by the decryption process. The outcomes are depicted in Figure 8. The test outcomes reveal that the information remains clear, demonstrating the algorithm’s effectiveness in resisting noise attacks and restoring ciphertext images.

#### 4.6.2. Cropping Attack Analysis

**Figure 9:**
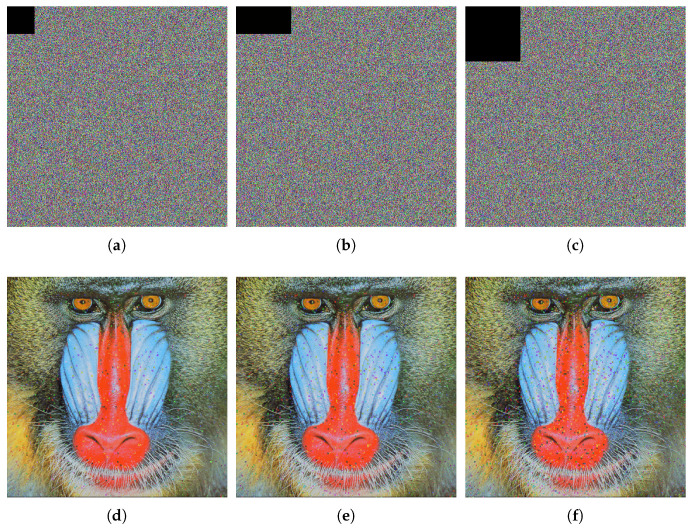
Cropping attack test for ciphertext image of 4.2.03. (**a**) Cropping 64×64 ciphertext image. (**b**) Cropping 64×128 ciphertext image. (**c**) Cropping 128×128 ciphertext image. (**d**) Decrypted image of (**a**). (**e**) Decrypted image of (**b**). (**f**) Decrypted image of (**c**).

During decryption, the ciphertext image undergoes varying levels of information loss, followed by the decryption operation. The outcomes are depicted in Figure 9. The experimental test demonstrates that the information remains clear, affirming the algorithm’s capability to effectively withstand cropping attacks and restore the ciphertext image.

### 4.7. Key Sensitivity Analysis

In this section, we explore encryption keys in detail and evaluate scheme performance. The security of a scheme is directly related to the property that minor adjustments to the key during encryption produces an entirely distinct result. This further emphasizes the complexity of evaluating the performance of security schemes, not only regarding the increase in encryption strength but also in terms of their sensitivity to input parameters. In the experiments, the original key and the scrambled key (original key+$10^{-14}$) are applied to encrypt a plaintext image. By calculating and comparing the NPCR and UACI between the ciphertext images obtained in the two cases, we were able to quantitatively assess the impact of minor variations in the key on the encryption results. Table 9 shows that the test data for the NPCR and UACI between the original ciphertext image and the scrambled ciphertext image meet the criteria, attesting to the robust key sensitivity of the CIEA-RQ; the test image is 4.2.03.

### 4.8. Key Space

All the parameters applied to produce the key make up the key space. Whether the key space is large enough is one of the crucial factors in measuring the security of an encryption scheme. In the CIEA-RQ, we adopt a quantum Logistic map as the mechanism for key generation. Its key space can be represented as *K* = {$x^{\prime }$, $y^{\prime }$, $z^{\prime }$, $r^{\prime }$, $\beta^{\prime }$}, where $x^{\prime },y^{\prime },z^{\prime },r^{\prime },\beta^{\prime }$ are keys with an accuracy of $10^{-14}$, and SHA-256 is the introduced hash function, which generates hash values of 256-bit length. The key space consisting of system parameters and the hash value is calculated to encompass approximately $2^{489}$ possible key combinations, with a key length of 489 bits. In consequence, the CIEA-RQ yields a considerable key space able to withstand exhaustive attacks. The comparison of the key space is presented in Table 10.

### 4.9. Efficiency Analysis

An effective encryption algorithm should not only ensure image security but also maintain high encryption efficiency to meet practical application demands. For the proposed CIEA-RQ, the average encryption time for three color images (4.1.03, 4.1.04, and 4.1.05) is 1.646906 s. Table 11 presents a comparison of encryption times between CIEA-RQ and other existing algorithms. As shown, CIEA-RQ requires less time, demonstrating superior encryption efficiency while maintaining strong security, making it more suitable for practical application scenarios.

## 5. Conclusions

In the algorithm, to achieve a balance between encryption efficiency and strength, the color plaintext image is segmented into bit planes. Based on different weights of information, encryption with varying complexity is applied to the high 4-bit planes and the low 4-bit planes separately. Then, cross-iterative diffusion is performed. Finally, the processed planes are combined to synthesize the color ciphertext image. RNA extended dynamic coding is used to encrypt high 4-bit planes, which improves the efficiency of encryption as well as significantly enhances the ability of the CIEA-RQ to resist cryptographic attacks. Meanwhile, the cross-iterative diffusion between the high 4-bit planes and the low 4-bit planes further confuses the encrypted result. This greatly increases the difficulty of cracking for attackers. The simulation outcomes reveal that the method possesses excellent robustness and the key space can go against brute-force attacks. NPCR and UACI test results show that the CIEA-RQ is resistant to differential attacks. The potential of the algorithm in other areas such as video and audio will be further explored in future research. In addition, we will deepen the analysis of the CIEA-RQ’s effectiveness and embed the system into practical applications to more comprehensively evaluate its performance in real-world scenarios.

## Figures and Tables

**Table 1:** RNA extended coding rules.

Rules	1	2	3	4	5	6	7	8	……	384
000	A	A	A	A	U	A	A	A	……	U
001	C	C	C	G	C	C	C	C	……	G
010	M	M	W	M	M	S	S	N	……	W
011	S	N	S	S	S	M	W	M	……	N
100	N	S	N	N	N	W	M	W	……	S
101	W	W	M	W	W	N	N	S	……	M
110	G	G	G	C	G	G	G	G	……	C
111	U	U	U	U	A	U	U	U	……	A

**Table 2:** RNA extended addition operation.

+	A	C	M	S	N	W	G	U
A	U	A	C	M	S	N	W	G
C	A	C	M	S	N	W	G	U
M	C	M	S	N	W	G	U	A
S	M	S	N	W	G	U	A	C
N	S	N	W	G	U	A	C	M
W	N	W	G	U	A	C	M	S
G	W	G	U	A	C	M	S	N
U	G	U	A	C	M	S	N	W

**Table 3:** RNA extended subtraction operation.

−	A	C	M	S	N	W	G	U
A	C	M	S	N	W	G	U	A
C	A	C	M	S	N	W	G	U
M	U	A	C	M	S	N	W	G
S	G	U	A	C	M	S	N	W
N	W	G	U	A	C	M	S	N
W	N	W	G	U	A	C	M	S
G	S	N	W	G	U	A	C	M
U	M	S	N	W	G	U	A	C

**Table 4:** RNA extended XOR operation.

⊕	A	C	M	S	N	W	G	U
A	W	N	U	G	C	A	S	M
C	N	W	G	U	A	C	M	S
M	U	G	W	N	S	M	C	A
S	G	U	N	W	M	S	A	C
N	C	A	S	M	W	N	U	G
W	A	C	M	S	N	W	G	U
G	S	M	C	A	U	G	W	N
U	M	S	A	C	G	U	N	W

**Table 5:** Adjacent pixel correlation test results.

File Name	Direction	Red		Green		Blue	
		Plaintext	Ciphertext	Plaintext	Ciphertext	Plaintext	Ciphertext
4.1.03	Horizontal	0.9296	−0.0061	0.9093	−0.0116	0.9252	0.0218
	Vertical	0.9789	0.0186	0.9749	0.0015	0.9723	−0.0086
	Diagonal	0.9073	0.0055	0.9011	0.0011	0.8974	−0.0008
	Anti-Diagonal	0.9229	−0.0080	0.9168	0.0254	0.8687	−0.0086
4.1.04	Horizontal	0.9892	0.0046	0.9825	−0.0086	0.9709	0.0261
	Vertical	0.9787	−0.0069	0.9676	0.0017	0.9473	−0.0059
	Diagonal	0.9725	0.0127	0.9470	0.0047	0.9332	−0.0018
	Anti-Diagonal	0.9705	−0.0038	0.9533	0.0113	0.9295	0.0093
4.1.05	Horizontal	0.9295	0.0003	0.9436	−0.0005	0.9729	0.0079
	Vertical	0.9626	−0.0113	0.9820	−0.0159	0.9794	0.0093
	Diagonal	0.9145	0.0089	0.9343	−0.0089	0.9573	0.0052
	Anti-Diagonal	0.9074	0.0013	0.9264	0.0002	0.9634	0.0223
4.2.03	Horizontal	0.8673	−0.0039	0.7631	−0.0073	0.8854	−0.0036
	Vertical	0.9200	−0.0006	0.8594	−0.0127	0.9093	0.0038
	Diagonal	0.8389	−0.0126	0.7504	0.0004	0.8383	0.0019
	Anti-Diagonal	0.8516	0.0004	0.7439	−0.0099	0.8321	0.0182
4.2.06	Horizontal	0.9519	0.0035	0.9717	−0.0092	0.9698	−0.0002
	Vertical	0.9574	0.0188	0.9700	0.0052	0.9717	0.0061
	Diagonal	0.9421	0.0016	0.9558	−0.0269	0.9555	0.0080
	Anti-Diagonal	0.9282	−0.0073	0.9525	0.0006	0.9525	0.0139
4.2.07	Horizontal	0.9700	−0.0133	0.9846	−0.0030	0.9676	0.0188
	Vertical	0.9640	−0.0035	0.9853	0.0355	0.9650	−0.0056
	Diagonal	0.9580	−0.0059	0.9737	−0.0056	0.9482	−0.0032
	Anti-Diagonal	0.9539	−0.0006	0.9708	−0.0001	0.9508	0.0032
2.2.01	Horizontal	0.9200	0.0243	0.9191	0.0186	0.9090	0.0049
	Vertical	0.9196	0.0027	0.9201	−0.0033	0.9129	0.0062
	Diagonal	0.8948	−0.0067	0.8981	−0.0074	0.8813	0.0084
	Anti-Diagonal	0.9000	−0.0049	0.8939	0.0080	0.8935	−0.0291
2.2.02	Horizontal	0.9331	−0.0256	0.8747	0.0035	0.7938	0.0016
	Vertical	0.9305	0.0071	0.8774	−0.0016	0.7798	0.0074
	Diagonal	0.9126	−0.0066	0.8298	0.0122	0.7306	−0.0039
	Anti-Diagonal	0.9126	0.0051	0.8465	−0.0011	0.7432	−0.0007
2.2.03	Horizontal	0.8920	−0.0059	0.8781	−0.0074	0.7630	−0.0096
	Vertical	0.9049	0.0099	0.8823	−0.0052	0.7846	−0.0211
	Diagonal	0.8328	0.0146	0.7945	−0.0001	0.6828	−0.0066
	Anti-Diagonal	0.8498	0.0033	0.8477	−0.0104	0.7286	−0.0058

**Table 6:** Adjacent pixel correlation of image in different algorithms.

Channel	Direction	CIEA-RQ	Ref. [39]	Ref. [40]	Ref. [41]
Red	Horizontal	−0.0039	0.0052	0.0003	0.0063
	Vertical	−0.0006	0.0043	0.0003	0.0024
	Diagonal	−0.0126	0.0032	0.0022	−0.0059
	Anti-Diagonal	0.0004	-	-	-
Green	Horizontal	−0.0073	0.0019	−0.0057	0.0132
	Vertical	−0.0127	0.0038	0.0121	0.0111
	Diagonal	0.0004	0.0024	−0.0144	−0.0131
	Anti-Diagonal	−0.0099	-	-	-
Blue	Horizontal	−0.0036	0.0018	0.0136	0.0126
	Vertical	0.0038	0.0045	0.0182	0.0108
	Diagonal	0.0019	0.0042	0.0027	−0.0007
	Anti-Diagonal	0.0182	-	-	-

**Table 7:** NPCR and UACI values.

Image Size	File Name	NPCR (%)			UACI (%)			Test Result
		Red	Green	Blue	Red	Green	Blue	
256×256×3	4.1.03	99.6155	99.6078	99.5926	33.4599	33.5099	33.4667	pass
	4.1.04	99.6063	99.6368	99.6124	33.4026	33.5585	33.3453	pass
	4.1.05	99.6201	99.5941	99.6231	33.3995	33.4350	33.3759	pass
512×512×3	4.2.03	99.6071	99.5922	99.6159	33.4525	33.4868	33.3868	pass
	4.2.06	99.6147	99.6140	99.6132	33.4150	33.5164	33.5318	pass
	4.2.07	99.6040	99.6029	99.5991	33.4987	33.5021	33.4993	pass
1024×1024×3	2.2.01	99.6035	99.6022	99.6052	33.4291	33.5016	33.4500	pass
	2.2.02	99.6062	99.6127	99.6046	33.4596	33.4751	33.4536	pass
	2.2.03	99.6184	99.6120	99.6152	33.4875	33.4474	33.4452	pass

**Table 8:** Image information entropy.

Image Size	File Name	Plaintext Image			Ciphertext Image		
		Red	Green	Blue	Red	Green	Blue
256×256×3	4.1.03	5.7150	5.3738	5.7117	7.9970	7.9974	7.9972
	4.1.04	7.2549	7.2704	6.7825	7.9970	7.9972	7.9973
	4.1.05	6.4311	6.5389	6.2320	7.9975	7.9973	7.9970
512×512×3	4.2.03	7.7067	7.4744	7.7522	7.9993	7.9993	7.9994
	4.2.06	7.3124	7.6429	7.2136	7.9994	7.9993	7.9993
	4.2.07	7.3388	7.4963	7.0583	7.9992	7.9993	7.9993
1024×1024×3	2.2.01	7.7575	7.3387	6.9561	7.9998	7.9998	7.9998
	2.2.02	6.0913	5.8474	4.6078	7.9998	7.9998	7.9998
	2.2.03	6.5140	5.7703	4.7036	7.9998	7.9998	7.9998

**Table 9:** Key sensitivity analysis.

Scrambled Key	NPCR (%)			UACI (%)			Result
	Red	Green	Blue	Red	Green	Blue	
$x^{\prime }+10^{-14}$	99.5926	99.6227	99.6040	33.4622	33.4975	33.5031	pass
$y^{\prime }+10^{-14}$	99.6010	99.6395	99.6239	33.3749	33.5214	33.5060	pass
$z^{\prime }+10^{-14}$	99.6128	99.6151	99.6040	33.4570	33.4016	33.4556	pass
$r^{\prime }+10^{-14}$	99.6136	99.6098	99.6170	33.4343	33.4665	33.4526	pass
$\beta^{\prime }+10^{-14}$	99.6197	99.6159	99.6056	33.4754	33.5267	33.4669	pass

**Table 10:** Key space analysis.

Encryption Scheme	CIEA-RQ	Ref. [39]	Ref. [43]	Ref. [44]	Ref. [45]	Ref. [46]
Key space	$2^{489}$	$2^{398}$	$2^{256}$	$2^{186}$	$2^{480}$	$2^{256}$

**Table 11:** Average encryption times (s) of different algorithms.

Encryption Scheme	CIEA-RQ	Ref. [47]	Ref. [48]	Ref. [49]
Time	1.646906	6.494822	1.855000	5.084900

## Data Availability

The datasets used and analyzed during the current study available from the corresponding author on reasonable request. All data generated or analyzed during this study are included in this article.
